# 2D nanosheets and composites for EMI shielding analysis

**DOI:** 10.1038/s41598-020-78614-6

**Published:** 2020-12-09

**Authors:** Ramsha Khan, Zeeshan Mehmood Khan, Hamza Bin Aqeel, Sofia Javed, Ahmed Shafqat, Ibrahim Qazi, Muhammad Abdul Basit, Rahim Jan

**Affiliations:** 1 School of Chemical and Materials Engineering (SCME), National University of Sciences and Technology (NUST), H-12, Islamabad, 44000 Pakistan; 2 Department of Materials Science and Engineering, Institute of Space Technology (IST), Islamabad, 44000 Pakistan; 3 Research Institute for Microwave and Millimeter-Wave Studies (RIMMS), National University of Sciences and Technology (NUST), H-12, Islamabad, Pakistan

**Keywords:** Graphene, Electronic properties and materials, Graphene, Two-dimensional materials

## Abstract

Liquid exfoliated, 2-dimensional (2D), few layered graphene and molybdenum disulfide nanosheets (GNS and MNS) are size selected for EMI shielding application. Scanning electron microscopy (SEM) has confirmed the lateral dimensions increase (1–2 µm for GNS and MNS) with lowering centrifugation speed (1000 to 500 rpm). The micron size (~ 15 µm) restacked structures of GNS and MNS (L ~ 2 µm) over a nylon membrane have shown ~ 16 dB and ~ 6 dB EMI shielding effectiveness (1–8 GHz frequency), respectively. The enhanced EMI shielding effectiveness for GNS-500 may be credited to its high carrier mobility as well as high aspect ratio of nanosheets. The GNS-500 are further dispersed (0.3 wt.%) in thermoplastic polyurethane for their applicability as flexible EMI shielding material. The dielectric characteristics predicted an enhancement for the attenuation (200 MHz–1 GHz). The experimental results (1–8 GHz) suggested the maximum attenuation ~ 18 dB showing the composite applicability as a broadband EMI shielding material.

## Introduction

Graphene experimental discovery in recent times has added a special interest in many applications due to its extraordinary set of properties, especially electrical and mechanical properties^1,2^. The zero bandgap, high carrier mobility (≈ 10,000 cm^2^ V^−1^ s^−1^) at room temperature and the Young’s modulus ~ 1 TPa along with strength ~ 130 GPa make it an excellent filler in polymer composites. Another two-dimensional (2D) material molybdenum disulphide (MoS_2_), a transition metal chalcogenide, is also evaluated due to its highly flexible properties in electronic and opto-electronic systems^3^. In comparison to graphene these materials show limited carrier mobility (200 cm^2^ V^−1^ s^−1^)^4^. However, the semi conductive behaviour along with hydrophilic nature and excellent mechanical properties make it a suitable candidate for dispersion in polymer composites as well. In polymers, both these 2D nanosheets, graphene and molybdenum disulphide may impart their intrinsic characteristics. One such characteristic is their electrical conductivity which is one of the main parameters for attenuation of electromagnetic interference (EMI). EMI has become a major concern with the rapid growth of electronic devices^5–8^. GNS and MNS based polymer composites, due to their ease of processability, light weight and cost effectiveness, may readily be utilized as EMI shielding materials. Graphene has been extensively used for EMI shielding applications^9–11^ while MoS_2_ has rarely been utilized^12,13^ in comparison with graphene. Mostly, it has been utilized in combination with reduced graphene oxide (rGO). Prasad et al. have attained SE ~ 4 dB (X-band) for MoS2/rGO, improved to 8 dB only with the addition of ferrites prepared using hydrothermal method^14^. In another work, MoS2/rGO system exhibited a higher SE ~ 16 dB which is enhanced to 20 dB when doped with gadolinium^15^. Here in this work, we have analysed liquid exfoliated, few layered, restacked 2D nanosheets of graphene and molybdenum disulphide individually for EMI shielding based on different aspect ratio. Graphene with high aspect ratio (GNS-500) showed higher attenuation (1–8 GHz) and was dispersed in thermoplastic polyurethane (TPU), a tough and flexible matrix. Dielectric characterisation provided an insight to TPU/GNS-500 sample utilization for EMI shielding application (1–8 GHz).

## Materials and methods

**Figure 1:**
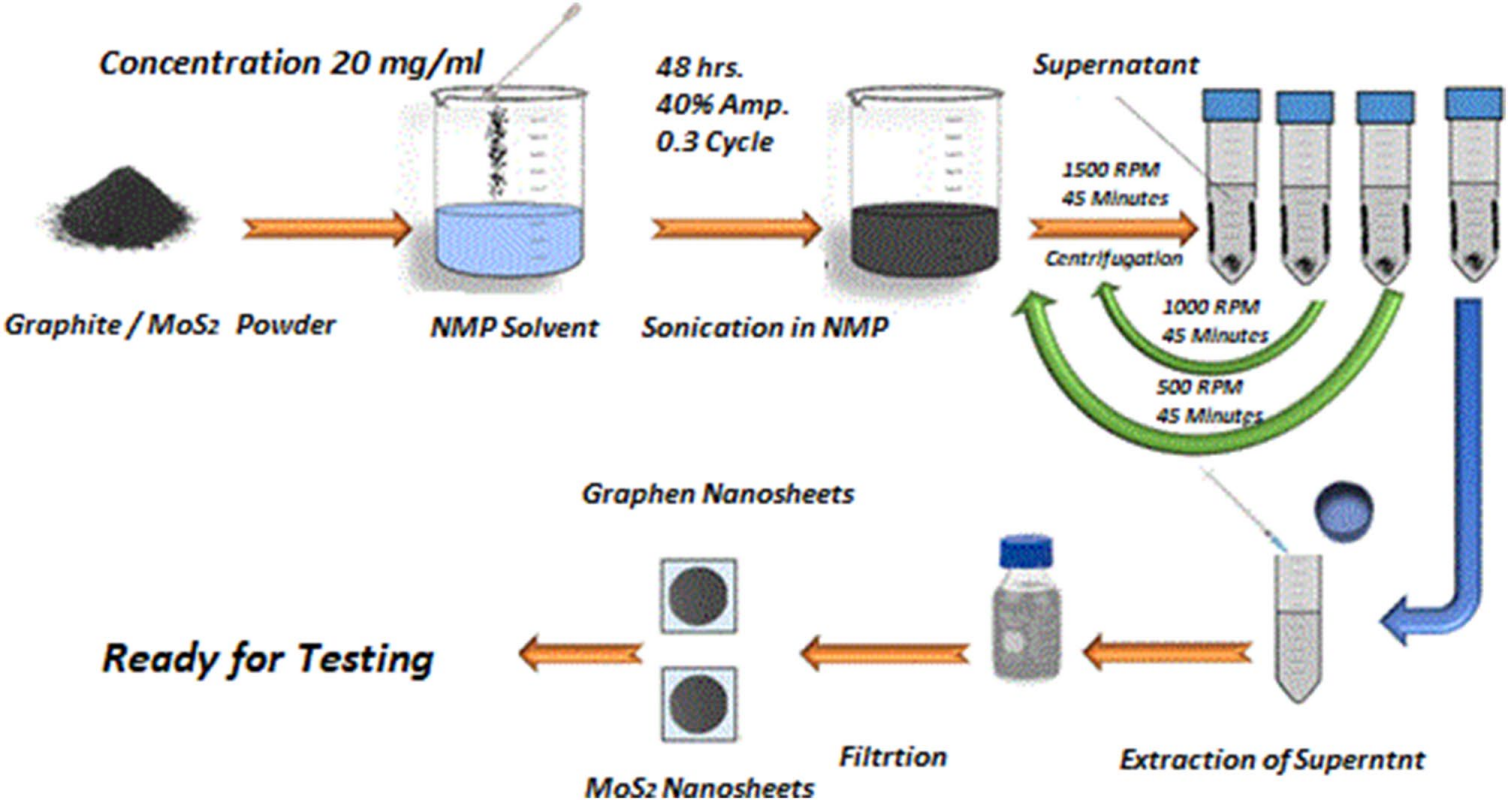
Schematic representation of Liquid Phase Exfoliation (LPE) method showing exfoliation of graphene and MoS_2_ powder at 500 and 1000 rpm.

Bulk MoS_2_ and graphite powder (purchased from China) are dispersed (20 mg/ml) in N-methyl-2-pyrrolidone (NMP). Both these dispersions are probe sonicated (48 h) using 40% amplitude at 0.3 cycle. Liquid phase exfoliation has emerged as a high yield production method for 2D nanosheets^16^. The exfoliated dispersions are then subjected to centrifugation (1500, 1000, 500 rpm—45 min each) for size selection. The size of the nanosheets is to increase with lowering centrifugation speed^17^. The relatively larger size selected dispersions of 1000 & 500 rpm are filtered out using nylon membrane (pore size: 0.45 microns) and dried (100 °C) afterwards in vacuum oven. The exfoliated, restacked nanosheets are dispersed in NMP (5 mg/ml) to prepare samples of GNS, MNS for EMI shielding effectiveness measurements. The dispersions of all the samples are deposited on to nylon films with a thickness around 15 µm. The schematic diagram is presented to elaborate the synthesis procedure (Fig. 1). Later, graphene is dispersed in tetrahydrofuran (THF) by mild bath sonication to mix in TPU/THF (50 mg/ml) solution at a 0.3 wt% concentration. The mixture is again bath sonicated (Branson 1510E-MT sonic bath) for 4 h. It is placed in vacuum oven for 24 h at room temperature followed by another period at 70 °C for 80 h to dry out THF and attain free standing composite film (0.5 mm) for EMI shielding effectiveness testing.

## Results and discussions

**Figure 2:**
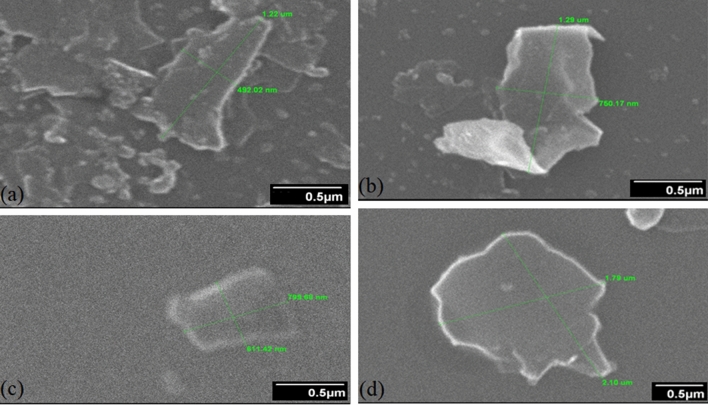
SEM representative images of exfoliated GNS (**a**) 1000 rpm, (**b**) 500 rpm and MoS2, (**c**) 1000 rpm, (**d**) 500 rpm.

**Figure 3:**
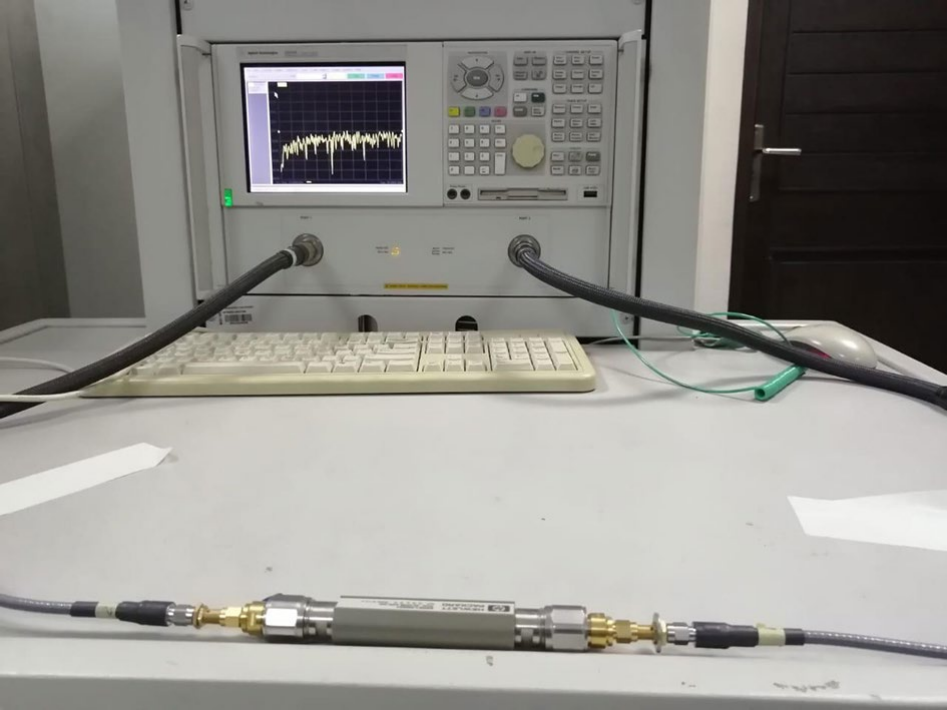
The vector network analyser test setup utilized for S-parameters measurements.

Liquid phase exfoliation (LPE) of 2D nanosheets and their utilization for EMI shielding application is the main course of this work. LPE helps in exfoliation of 2D materials up to 3–10 layers along with size selection^18^. State of exfoliation and dimensional analysis of nanosheets is estimated with scanning electron microscope (SEM). Scanning electron microscopy (SEM-JOEL JSM-6490A) gives an indication of the nanosheets lateral dimensions ‘L’. As per representative SEM micrographs (Fig. 2a–d), the lateral dimensions of both GNS and MNS are around ~ 1 µm at 1000 rpm. As per the representative images (Fig. 2a,c), the lateral dimension of GNS-1000 is ~ 1.22 µm while for MNS-1000 it is ~ 0.8 µm. At lower centrifugation (500 rpm), the lateral dimensions for both the nanosheets is enhanced which is ~ 2 µm for both GNS-500 and MNS-500 (Fig. 2 b, d). The marker showing 1.29 µm on GNS-500 (Fig. 2b) is misleading as one must consider the curving effect. The reported thickness of MoS_2_ and graphene monolayers is 0.65 nm^19^ and 0.35 nm respectively. Keeping in view the no. of layers to be around 3–10; the aspect ratio (L/t) for lower centrifugations (500 rpm) nanosheets is more in comparison with high centrifugation (1000 rpm) nanosheets. Further analysis with transmission electron microscope (TEM) and Raman spectroscopy may give a clearer estimation for the dimensions. All these samples, GNS and MNS (both 500 & 1000 rpms) are tested for initial estimation of EMI shielding effectiveness. The samples are prepared in toroid shape (3 mm inner, 7 mm outer diameter). EMI shielding effectiveness (SE) measurements are performed using vector network analyser system (Fig. 3) in 1–8 GHz frequency range^20^. Experimentally, EMI SE is measured in decibels (dB) as the logarithmic ratio of incoming power (P_I_) to transmitted power (P_T_). It can be written as^21^

1 $$SE\left(dB\right)=10log\frac{{P}_{I}}{{P}_{T}}$$.

When electromagnetic radiations are incident on a shielding device, the reflection (R), absorption (A), and transmission (T) must add up to 1, that is,

2 $$R+A+T=1$$.

The reflection (R) and transmission (T) coefficients values are obtained from the network analyser in form of scattering parameters i.e. S_11_, S_12_, S_21_, S_22_ which can be used to find R and T coefficients as:

3 $$R={\left|{S}_{11}\right|}^{2}={\left|{S}_{22}\right|}^{2}$$

4 $$T={\left|{S}_{12}\right|}^{2}={\left|{S}_{21}\right|}^{2}$$

The total EMI SE (SE_T_) is the sum of the contributions from reflection (SE_R_), absorption (SE_A_) and multiple internal reflections (SE_M_). At higher EMI SE values, and with a multilayer EMI shield, contribution from multiple internal reflection is merged in the absorption, because the re-reflected waves get dissipated in form of heat in the shielding material. The total SE_T_ can be written as^22,23^:

5 $${SE}_{T}={SE}_{R}+{SE}_{A}$$

**Figure 4:**
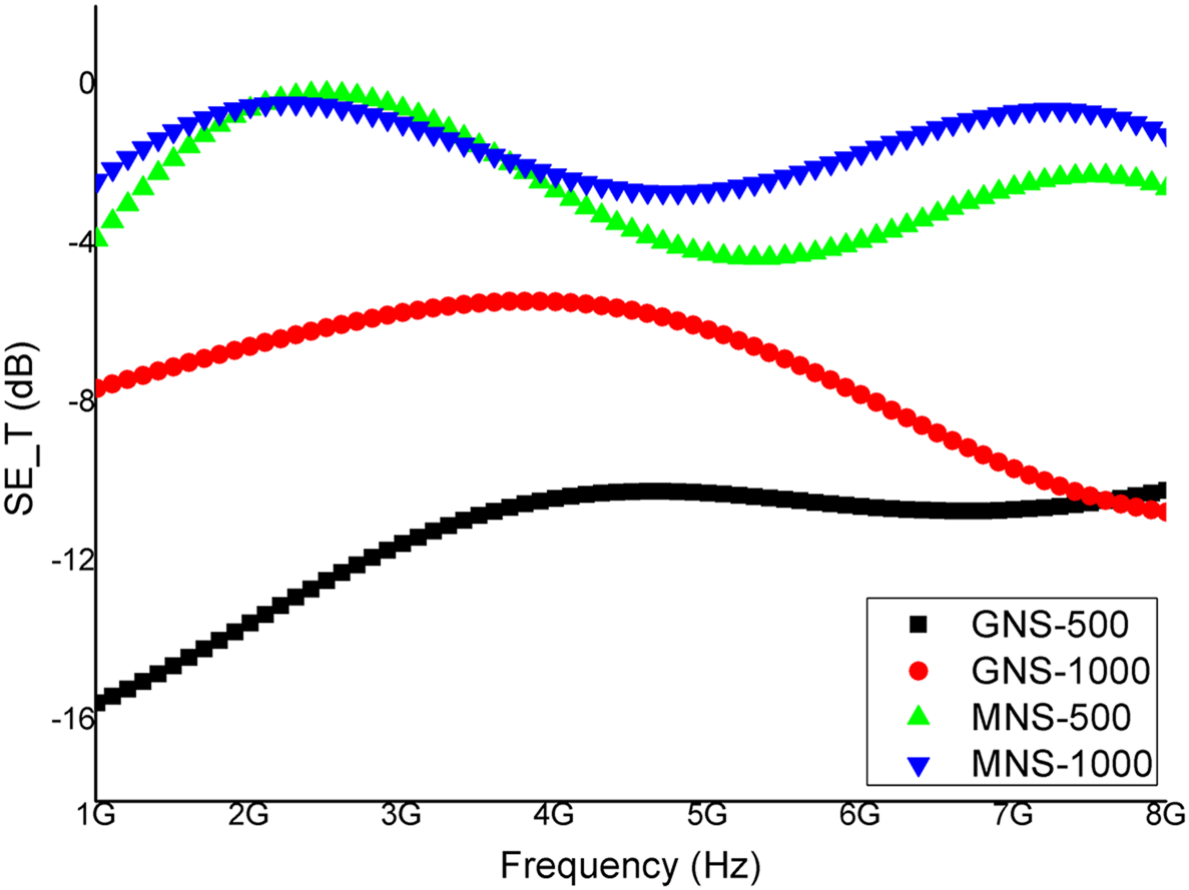
EMI shielding effectiveness analysis (experimental) of GNS and MNS both for 1000 rpm and 500 rpm as a function of frequency (1–8 GHz).

**Figure 5:**
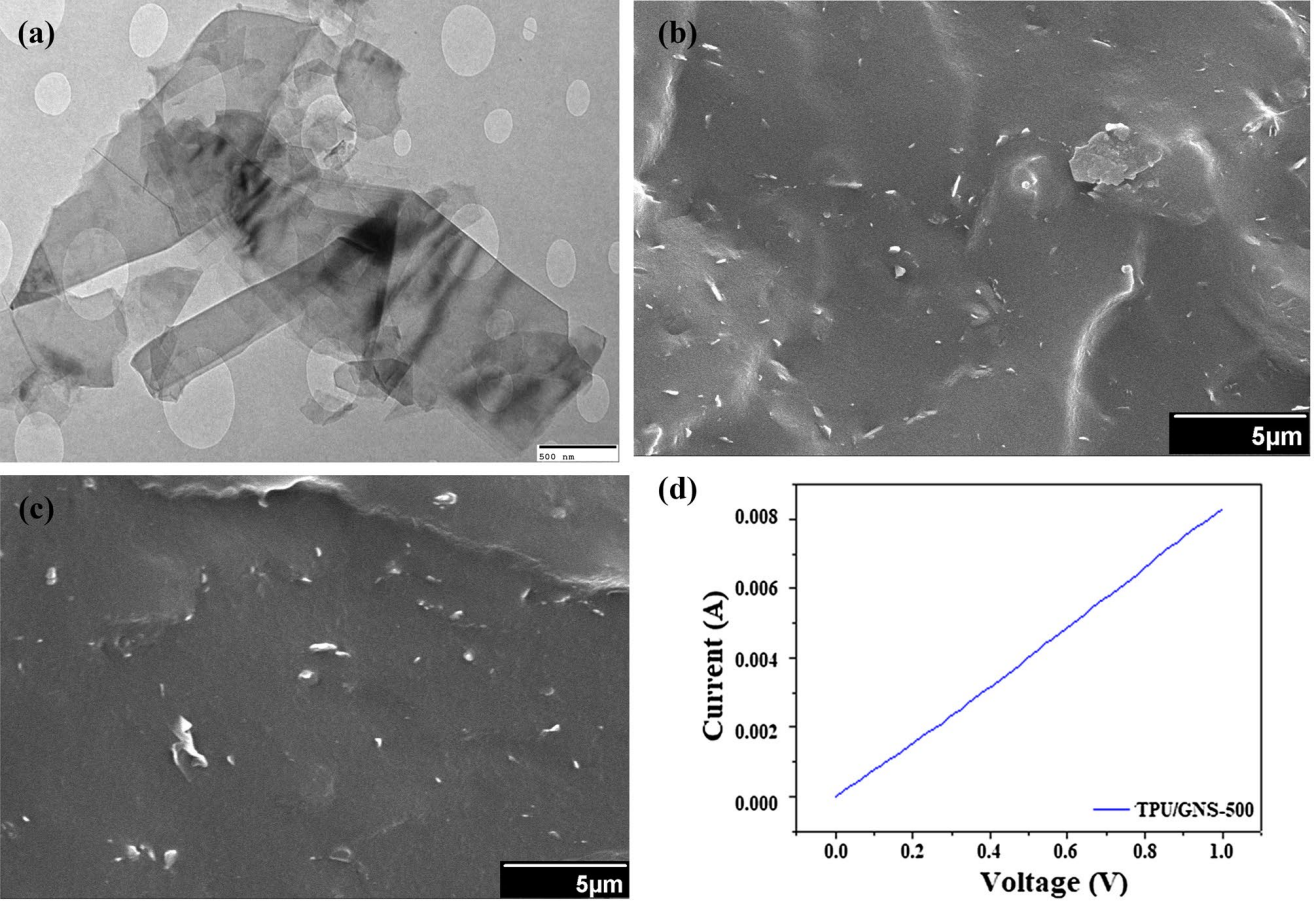
(**a**) GNS-500 representative TEM image showing few layered graphene nanosheets (**b**,**c**) Cross-sectional morphology (**d**) Electrical conductivity (DC) of TPU/GNS-500 composite.

**Figure 6:**
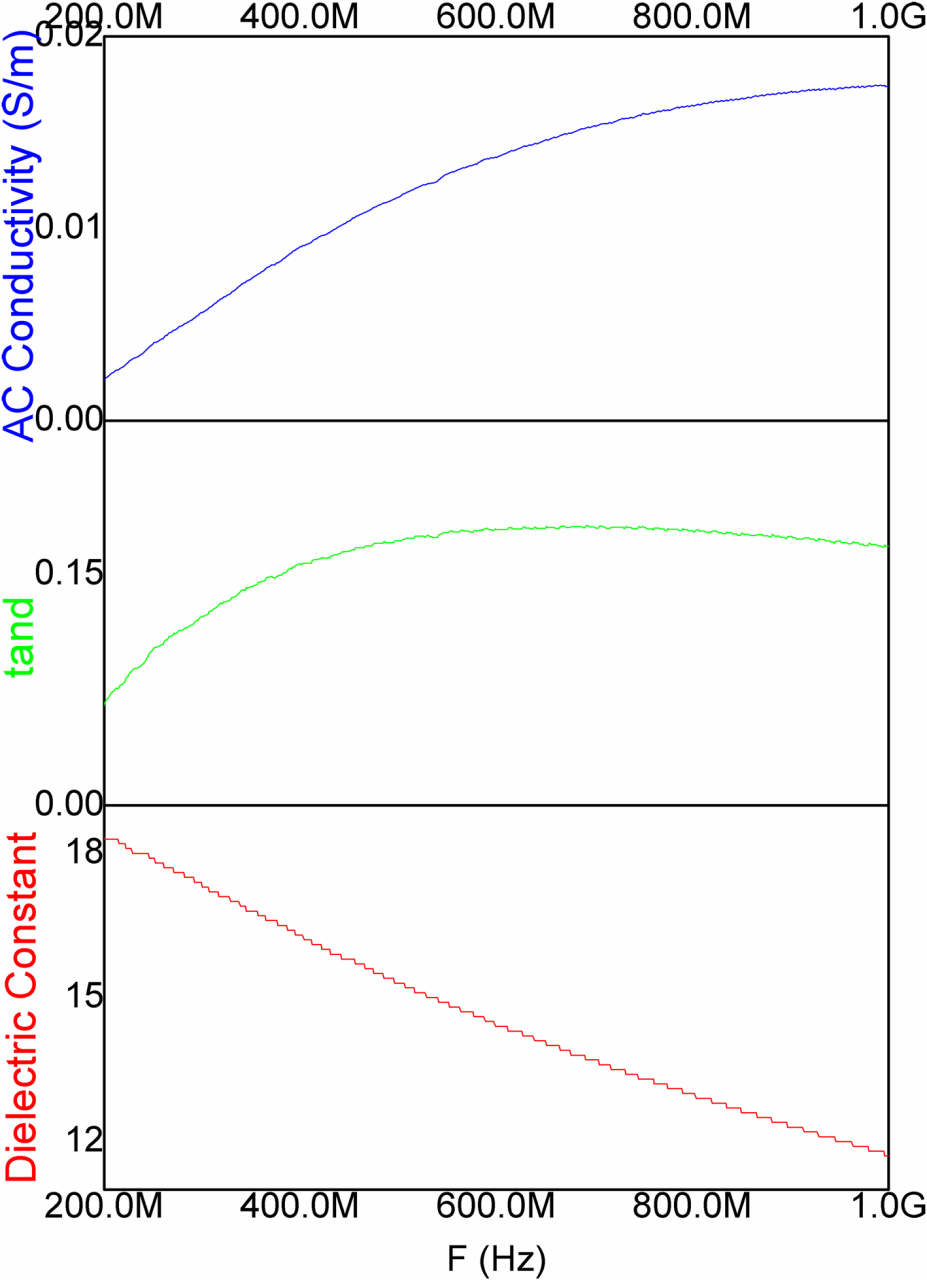
Dielectric constant, Tangent loss and AC conductivity measurements of TPU/GNS-500 composite.

**Figure 7:**
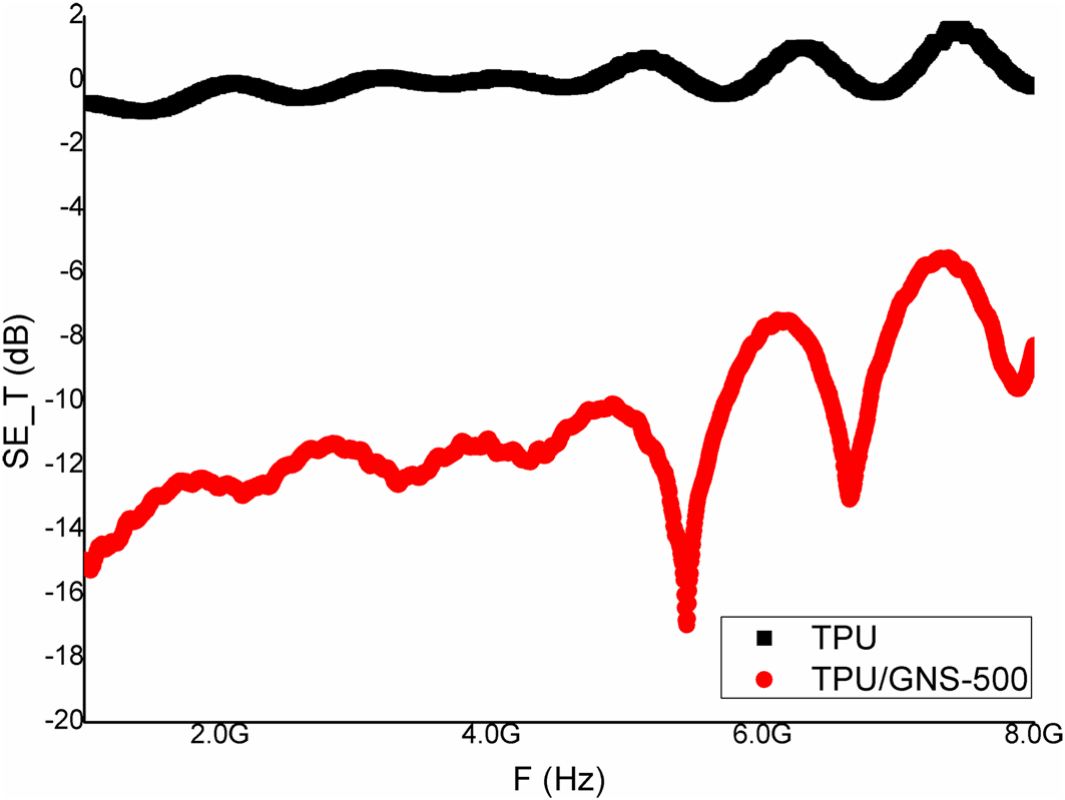
Experimental EMI shielding effectiveness results for TPU and TPU/GNS-500 Composite based on s-parameters as function of frequency (1–8 GHz).

The results for EMI SE are shown in Fig. 4. The high aspect ratio of graphene sheets tends to raise the percolation network in the nylon films due to their high surface area and hence, high electrical conductivity along with enhanced EMI shielding effectiveness. The effect is clearly seen for GNS-500 and GNS-1000 in Fig. 4 where the former has a maximum SE value ~ 16 dB at lower frequencies (1–2 GHz) and remains throughout ~ 12 dB. For GNS-1000, the SE_T_ is around 8–12 for the tested frequency range. In case of MNS, the SE_T_ response is same as for the aspect ratio consideration in relation to GNS. MNS-500 has maximum SE_T_ ~ 5 dB as compared ≤ 3 dB for MNS-1000 at 5–6 GHz while below this frequency the SE_T_ response is almost same. Although, 5 dB attenuation (MNS) is lower but with functionalization process it may be enhanced in future work. In current work graphene possessing higher charge carrier mobility, the main reason for higher attenuation, has been utilized for free standing TPU based composite films. Transmission electron microscopy (TEM) representative image (Fig. 5a) confirms the few layered nanosheets. The cross-sectional morphology of TPU/GNS-500 composite depict the homogeneous dispersion of nanosheets inside the polymer matrix as shown in Fig. 5b,c. Electrical conductivity is one of the main parameters in case of EMI shielding effectiveness. Figure 5d shows I–V trend, needed to determine DC conductivity of TPU/GNS-500 composite, confirms its conducting network formation. The DC conductivity of the TPU/GNS-500 is ~ 1.02 S/m, recorded using Kiethley 2420 source meter in the range of 0 to 10 V. The above-mentioned DC conductivity along-with the frequency dependent AC conductivity (derived from the dielectric nature of material) indicates the material’s applicability for EMI shielding. The TPU/GNS-500 composite is evaluated experimentally (200 MHz–1 GHz) for the dielectric characteristics (Wayne Kerr Electronic Equipment). The composite sample (8 mm diameter) is placed in between copper rods, with smooth surfaces, arranged as parallel plates capacitor formation. Using capacitance and tangent loss which are measured directly, other parameters like dielectric constant and AC conductivity were derived as shown in Fig. 6. The dielectric constant has a decreasing trend as a function of frequency while the dielectric loss incremental behavior is clearly translated into the AC conductivity shown in Eq. (6):

6 $${\sigma }_{AC}=\omega {\varepsilon }_{o}{\varepsilon }^{^{\prime}}tan\delta$$

where ‘*ω*’ is the angular frequency, *ε*_*o*_ is the permittivity of free space, *έ* shows the dielectric constant and *tanδ* is the ratio of dielectric loss and dielectric constant. The dielectric constant, depicting the material’s ability to store energy, ranges from 12–18. While the *tanδ* is the measure of dissipation energy indicative of various losses inside an oscillating field. Both the mentioned parameters are utilized to measure the AC conductivity, which is increasing with the frequency and reaches up to 0.02 S/m around 1 GHz. The voltage applied for dielectric measurements is in milli volts (mV) which may have caused the lower AC conductivity response in comparison to DC conductivity. Dielectric characteristics provide an insight showing TPU/GNS-500 may be utilized for EMI shielding application. Therefore, the experimental analysis was carried out in 1–8 GHz as was done for the GNS and MNS only samples. The EMI shielding effectiveness (SE) for TPU/GNS-500 is calculated using Eqs. (1) – (5). The insulating nature of polymer is evident from the EMI SE graph (Fig. 7) which shows ~ 0 dB attenuation. In case of composite, graphene’s conducting nature has been imparted to the otherwise insulating polymer (TPU). The EMI SE is ~ 16 dB at 1 GHz which reaches to maximum value of ~ 18 dB around 5–6 GHz frequency regime. It is in continuation with the dielectric characteristics in the frequency range 200 MHz—1 GHz. The experimental evaluation of the TPU/GNS-500 composite reveals that with an addition of only 0.3 wt.% graphene, a free standing, broad band EMI shielded material has been fabricated. Electrical conductivity and dielectric dissipation are the main parameters responsible for the attenuation. The shielding effectiveness may further be enhanced if the magnetic fillers are added, as both electric and magnetic dissipation of electromagnetic waves add up for EMI shielding phenomena. It will also boast the almost negligible absorption shielding effectiveness in present case, as the reflection based shielding effectiveness is dominant. The work in its initial stage, shows promising results to be taken up further to the levels of application in the form of coatings comprising of various other 2D nanosheets for EMI shielding and electrical sensing applications.

## Conclusions

Liquid phase exfoliated graphene and molybdenum disulphide nanosheets (GNS and MNS) have been ranked via aspect ratio for an initial assessment of EMI shielding effectiveness (1–8 GHz). GNS-500 has shown ~ 16 dB attenuation (experimental) as compared to MNS-500 ~ 5 dB shielding effectiveness mainly due to high carrier mobility of graphene. The GNS-500 are dispersed in thermoplastic polyurethane (TPU) and evaluated for DC (~ 1.02 S/m) and AC conductivity (0.02 S/m at 1 GHz) along with high dielectric constant (12–18) & tangent loss (~ 0.15). Experimental measurements have proved the broadband effectiveness (18 dB max.) for EMI shielding. The high aspect ratio nanosheets of various graphene like 2D materials may further be exploited further for EMI shielding, electrical sensing and storage applications.
